# The First Mitochondrial Genomes of the Family Haplodiplatyidae (Insecta: Dermaptera) Reveal Intraspecific Variation and Extensive Gene Rearrangement

**DOI:** 10.3390/biology11060807

**Published:** 2022-05-25

**Authors:** Hong-Ling Liu, Song Chen, Qing-Dong Chen, De-Qiang Pu, Zhi-Teng Chen, Yue-Yue Liu, Xu Liu

**Affiliations:** 1 Key Laboratory of Integrated Pest Management on Crops in Southwest, Institute of Plant Protection, Sichuan Academy of Agricultural Sciences, Ministry of Agriculture, Chengdu 610066, China; liuhongling1972@outlook.com (H.-L.L.); chensong1980@outlook.com (S.C.); chenqingdong03@outlook.com (Q.-D.C.); pdqpudeqiang@163.com (D.-Q.P.); 2 School of Grain Science and Technology, Jiangsu University of Science and Technology, Zhenjiang 212004, China; chenzhiteng@just.edu.cn; 3 Analysis and Testing Center, Sichuan Academy of Agricultural Sciences, Chengdu 610066, China; liuyueyue1991@outlook.com

**Keywords:** earwigs, Haplodiplatyidae, mitogenomes, gene rearrangement, phylogeny

## Abstract

**Simple Summary:**

The insect order Dermaptera is commonly known as earwigs. The earwigs have many interesting biological characteristics, such as epizoic on other small animals, viviparous, and maternal care on their eggs and young nymphs. The external morphology of earwigs has been studied in detail, but their genetic characteristics remain unclear. The phylogenetic position of Dermaptera among all insect orders and the inner relationship of Dermaptera are largely unsolved. To better understand the molecular characters of earwigs, we sequenced and analyzed two mitogenomes of an earwig species from the family Haplodiplatyidae. The results revealed the existence of intraspecific variation and extensive gene rearrangement events in the mitogenomes of earwigs. The phylogenetic results are partially similar to previous studies. The discoveries in this study could provide new information for the molecular diversity and mitogenomic evolution of earwigs.

**Abstract:**

Haplodiplatyidae is a recently established earwig family with over 40 species representing a single genus, *Haplodiplatys* Hincks, 1955. The morphology of Haplodiplatyidae has been studied in detail, but its molecular characters remain unclear. In this study, two mitogenomes of *Haplodiplatys aotouensis* Ma & Chen, 1991, were sequenced based on two samples from Fujian and Jiangxi provinces, respectively. These represent the first mitogenomes for the family Haplodiplatyidae. The next-generation sequencing method and subsequent automatic assembly obtained two mitogenomes. The two mitogenomes of *H. aotouensis* were generally identical but still exhibit a few sequence differences involving protein-coding genes (PCGs), ribosomal RNA (rRNA) genes, control regions, and intergenic spacers. The typical set of 37 mitochondrial genes was annotated, while many transfer RNA (tRNA) genes were rearranged from their ancestral locations. The calculation of nonsynonymous (Ka) and synonymous (Ks) substitution rates in PCGs indicated the fastest evolving *nd4l* gene in *H. aotouensis*. The phylogenetic analyses supported the basal position of Apachyidae but also recovered several controversial clades.

## 1. Introduction

The hemimetabolous insect order Dermaptera, commonly known as earwigs, is a relatively small and primitive group of terrestrial insects comprising over 1900 extant species worldwide [1,2]. The earwigs can be easily distinguished from other insects in the adult stage by the pincer-like, unsegmented cerci, which have various functions in defense, prey capture, wing folding, and mating [3,4]. Most earwigs are free-living and oviparous, while a few species are epizoic on vertebrate hosts and viviparous [5]. The earwigs mainly feed on plant material, whereas some groups are predators or scavengers. The females of earwigs will protect their eggs from the attack of predators and clean the eggs with their mouthparts; they also feed the first-instar nymphs [6,7].

The monogeneric family Haplodiplatyidae was established based on the genus *Haplodiplatys* Hincks, 1955, which was originally included in another family, Diplatyidae [8,9]. The paraphyly of Diplatyidae *sensu lato* (including current Diplatyidae and Haplodiplatyidae) has already been proposed by earlier morphological studies [4,10,11,12]. The genus *Haplodiplatys* comprises over 40 species, one of which was described from Miocene Mexican amber [2,13,14]. The species *Haplodiplatys aotouensis* Ma & Chen, 1991, was originally described from Fujian Province of southeast China [15] and was later included in the monograph of Chen & Ma (2004) [16]. No related studies were conducted for *H. aotouensis* since then, however, its occurrence in neighboring provinces is continuously witnessed by insect collectors.

Despite the efforts using either morphological or molecular data, the phylogenetic position of Dermaptera and the inner relationships between dermapteran taxa remain largely unsolved [17,18,19,20]. Research on the genetic characters of earwigs is relatively weaker than traditional morphological studies. Research of the widely used genetic marker, mitochondrial genome (mitogenome), is rather little in Dermaptera when compared with other insects [21]. To date, the complete or nearly complete mitogenomes of only six species have been sequenced and analyzed (Table 1) [17,20]. Some common mitogenomic characteristics of earwigs and the preliminary phylogenetic results are summarized in Chen (2022) [20] but require further confirmation with more mitogenomic data. In this study, we sequenced and analyzed the mitogenomes of *H. aotouensis* based on samples collected from two different geographic locations. The study aims to investigate the intraspecific mitogenomic variation, the common mitogenomic structural characters, and the phylogenetic relationships of earwigs.

## 2. Materials and Methods

### 2.1. Insect Collection and DNA Extraction

Two samples of *H. aotouensis* were respectively collected from Wuyishan Natural Reserve (27.7464° N, 117.6838° E), Fujian Province, in March of 2022, and Wuyi Mountain (27.9803° N, 117.78071894° E), Jiangxi Province, in May of 2021. The specimens were identified as *H. aotouensis* by the author. The collected specimens were preserved in 100% ethanol until used for the total genomic DNA extraction by E.Z.N.A. Tissue DNA Kit (Omega Bio-Tek, Inc., Norcross, GA, USA).

### 2.2. Mitogenome Sequencing and Assembly

The TruSeq DNA Library (insert size = 400 bp) was constructed using at least 1 μg of DNA according to standard protocols. The library was sequenced by Illumina HiSeq 4000 platform (Nanjing Personal Gene Technology Co., Ltd., Nanjing, China) with paired-end reads of 2 × 150 bases. Clean reads were obtained by removing unpaired, short, and low-quality raw reads. The high-quality reads were assembled by the GetOrganelle pipeline v 1.7.4 [24]. The two mitogenomes were deposited in GenBank under the accession numbers ON186792 and ON186793.

### 2.3. Mitogenome Annotation and Analysis

The MITOS online server was used to annotate the assembled mitogenomes and predict the secondary structure of the tRNA genes [25]. The annotation results were validated and corrected by homology alignments with other earwigs and the NCBI’s ORF Finder (https://www.ncbi.nlm.nih.gov/orffinder/, accessed on 1 May 2022). The mitogenome structure and GC skews were visualized by the CGView Server (http://stothard.afns.ualberta.ca/cgview_server/, accessed on 1 May 2022) [26]. MEGA-X was used to calculate nucleotide composition, codon usage, and relative synonymous codon usage (RSCU) [27]. The composition skew values in the mitogenome were calculated using the following formulas [28]: AT-skew = (A − T)/(A + T) and GC-skew = (G − C)/(G + C). The probable mitochondrial rearrangement scenarios during the evolution of *H. aotouensis* and other earwigs were predicted by CREx (Common Interval Rearrangement Explorer) online server [29] using *Drosophila yakuba* Burla, 1954 as a reference [30,31]. The synonymous substitution rate (Ks) and nonsynonymous substitution rate (Ka) were calculated using KaKs_Calculator v 2.0 [32]. Tandem repeats in the mitogenome were identified using the online tool Tandem Repeats Finder (http://tandem.bu.edu/trf/trf.advanced.submit.html, accessed on 1 May 2022) [33].

### 2.4. Phylogenetic Analysis

The mitogenome sequences of the other six species of Dermaptera and an outgroup from Plecoptera were downloaded from GenBank and used in the phylogenetic analysis (Table 1). The nucleotide sequences of PCGs were aligned by MUSCLE in MEGA X with default settings of codon mode [34,35] and manually trimmed for length consistency. DAMBE 6.4.42 [36] was used to calculate the substitution saturation of the two rRNA genes and each codon position of the PCGs under the GTR model or F84 model when an error occurs. Due to the lack of several PCGs in *Diplatys flavicollis* Shiraki, 1907 (Diplatyidae), and the results of substitution saturation plots, *atp8*, *cox1*, *cox2*, *nad2*, *nad4l*, and *nad6*, the third codon positions of the other seven PCGs, and the two rRNA genes were excluded from the nucleotide dataset. The first two codon positions of the remaining seven PCGs were concatenated into a combined nucleotide dataset using SequenceMatrix v 1.7.8 [37]. Another amino acid dataset was established by translation of the concatenated nucleotide sequence of the seven PCGs, including all codon positions. PartitionFinder v 2.1.1 was used to evaluate the best substitution models and partitioning schemes for the nucleotide dataset with the Bayesian Information Criterion (BIC) and a greedy search algorithm [38]. Each dataset was used to conduct three phylogenetic inferences, including Phylo–Bayesian (PB) inference, Bayesian inference (BI), and maximum likelihood (ML) analysis. PB inferences for both datasets were performed with the site-heterogeneous model CAT + GTR (two independent chains, constant sites removed, gamma-distributed rates with four categories) implemented in Phylobayes v 3.3 [39]. MrBayes v 3.2.7 was employed to construct the BI trees for both datasets, running four independent Markov chains for 20 million generations and sampled every 1000 generations [40]. A burn-in of 25% was used to generate the consensus tree. RAxML v 8.2.12 was used to construct the ML trees for both datasets, with 1000 bootstrap replicates [41]. FigTree v 1.4.2 was employed to edit and visualize the phylogenetic trees.

## 3. Results

### 3.1. Mitogenome Structure and Nucleotide Composition

**Figure 1:**
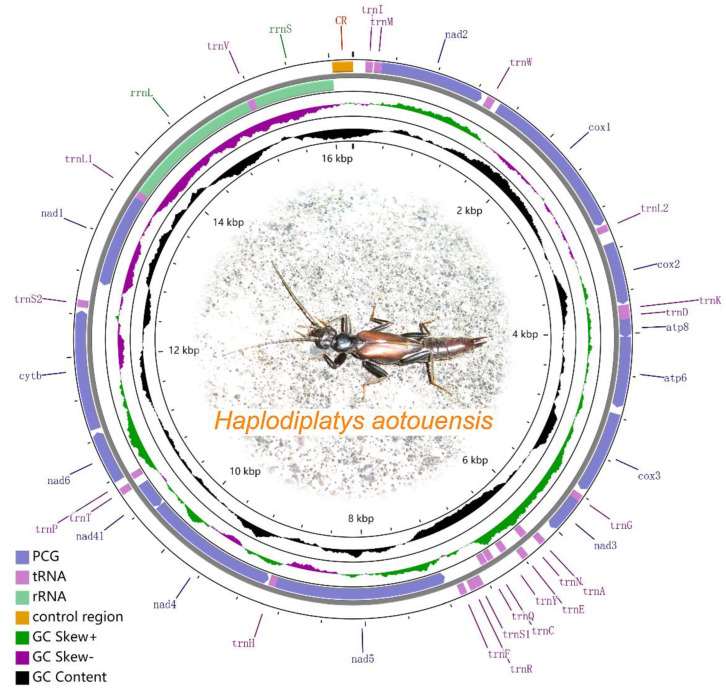
Consensus mitogenome structure of *Haplodiplatys aotouensis*. The map is generated based on Fujian’s sample. Genes outside the map are transcribed clockwise, while those inside the map are transcribed counterclockwise. Names and other details of the genes are listed in Table 2. The inside circles show the GC content and the GC skew. GC content and GC skew are plotted as the deviation from the average value of the entire sequence.

The two mitogenomes obtained from two geographic areas are highly identical (99.4%), which confirms both to be the same species (Figure 1). The *H. aotouensis* mitogenome from Fujian Province is 16,134 bp long whereas the mitogenome from Jiangxi Province is 16,222 bp in length (Table 2). Both mitogenomes consist of the standard set of 37 genes (13 PCGs, 22 tRNA genes, and two rRNA genes), however, differ in the following aspects: *cox1*, *cox2*, and *nad6* genes each have one different base on position 951, 300, and 384, respectively; *rrnS* gene of Jiangxi’s sample has one additional base in the middle section than Fujian’s sample; length difference of four intergenic regions; length difference of the control regions (Table 2). The majority J-strand had 24 genes and the minority N-strand has the remaining 14 genes. In the mitogenomes of *H. aotouensis*, a total of four overlapping regions were found, ranging in size from 1 to 7 bp. The longest overlapping region was found between *atp8* and *atp6* (Table 2). There are also 23 intergenic spacers ranging in size from 2 to 178 bp and the longest gene spacer was located between *trnS2* and *nad1* (Table 2).

The two *H. aotouensis* mitogenomes are highly skewed towards A and T nucleotides, with an A + T content of 71.7% in Fujian’s sample and 71.9% in Jiangxi’s sample. Each of the 37 mitochondrial genes also has a rich A + T content ranging from 63.6% in *trnK* to 86.6% in *trnC* (Table 2). The AT skew is negative (−0.1), whereas the GC skew is positive (0.3) in both mitogenomes.

### 3.2. Gene Rearrangement

**Figure 2:**
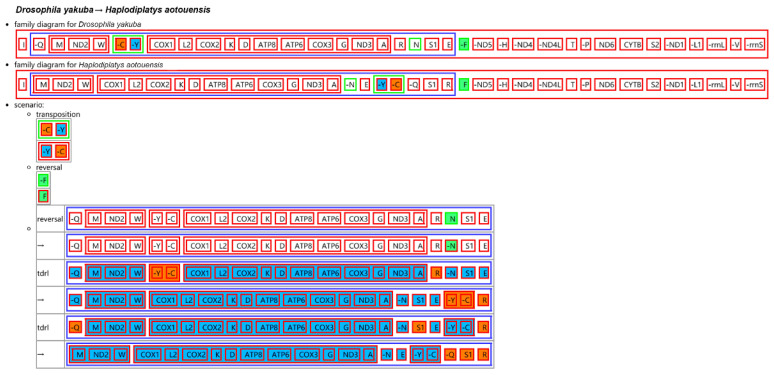
Reconstruction of mitochondrial gene rearrangement scenarios in the evolution of *Haplodiplatys aotouensis*. The ancestral mitochondrial gene arrangement of *Drosophila yakuba* is set as the reference. The tRNA genes are represented by the amino acid abbreviations. In each rearrangement event of the scenario, the upper box indicates the earlier arrangement of related genes, whereas the lower box indicates the rearranged gene order after the event.

The gene order of the 13 PCGs of the two *H. aotouensis* mitogenomes is identical and conserved with all sequenced earwigs and the presumed ancestral arthropod mitochondrial gene arrangement of *D. yakuba* [20,31]. However, tRNA genes in the three gene clusters, *trnI-Q-M*, *trnW-C-Y*, and *trnA-R-N-S1-E-F* are rearranged, and such rearrangement pattern is not found in any sequenced earwigs [20]. The CREx analysis demonstrated that the gene order of the *H. aotouensis* mitogenomes is rearranged from the ancestral type of mitogenome of *D. yakuba* by the following steps (Figure 2): initial transposition of *trnC* and *trnY*; subsequent reversal of *trnF* and *trnN*; and two final tandem duplication and random loss (TDRL) events regarding *trnY-trnC*, *trnR*, *trnQ*, and *trnS1*.

### 3.3. PCGs

**Figure 3:**
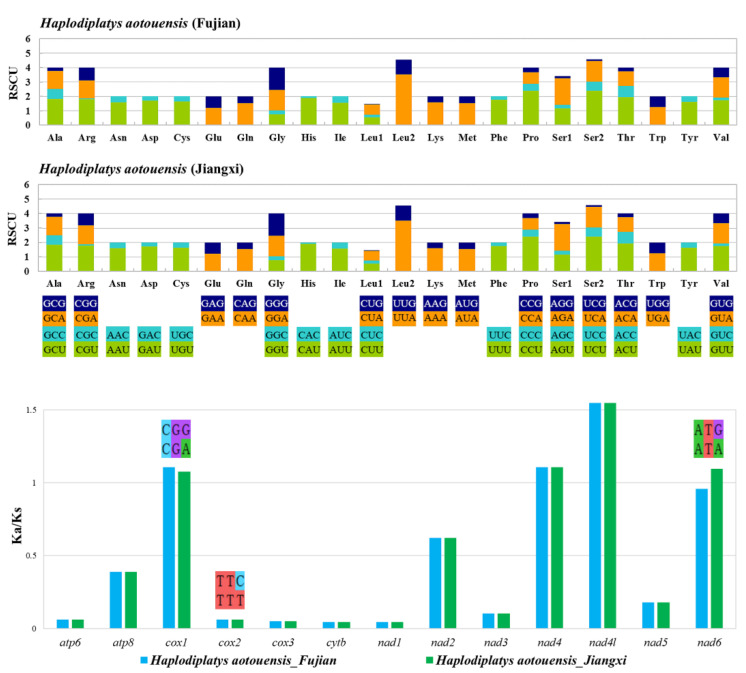
Relative synonymous codon usage (RSCU) and evolutionary rates of PCGs in *Haplodiplatys aotouensis*. In the Ka/Ks chart, t1he different codons between Fujian’s (**upper**) and Jiangxi’s (**lower**) samples are marked above the relevant PCGs.

The 13 PCGs of *H. aotouensis* are similar in size to those of other sequenced earwigs [20]. All PCGs start with the standard ATN start codons (ATT, ATC, and ATG) and terminate with the complete stop codon TAN (TAA or TAG). The codon usage of PCGs was assessed by the relative synonymous codon usage (RSCU) value, which indicates the number of times a codon is repeated in relation to the uniform synonymous codon usage (Figure 3). Among the amino acid-encoding codons, TTA (Leu), TCT (Ser), and CCT (Pro) are the most frequently used. The ratio of Ka/Ks for each PCG was calculated to assess their evolutionary rates (Figure 3). The results indicate that *nad4l* has the highest evolutionary rate, followed by *cox1*, *nad4*, and *nad6*. The Ka/Ks ratios of *cox1*, *cox2*, and *nad6* differ between the two geographical samples of *H. aotouensis*. The calculation also reveals a slightly higher evolution rate in *cox1* and *cox2* of Fujian’s sample and *nad6* in Jiangxi’s sample. The ratios of four genes are above 1, indicating their evolution under positive selection. The remaining nine PCGs show much lower Ka/Ks ratios below 1, suggesting the existence of purifying selection in these PCGs.

### 3.4. tRNAs, rRNAs and the Control Region

**Figure 4:**
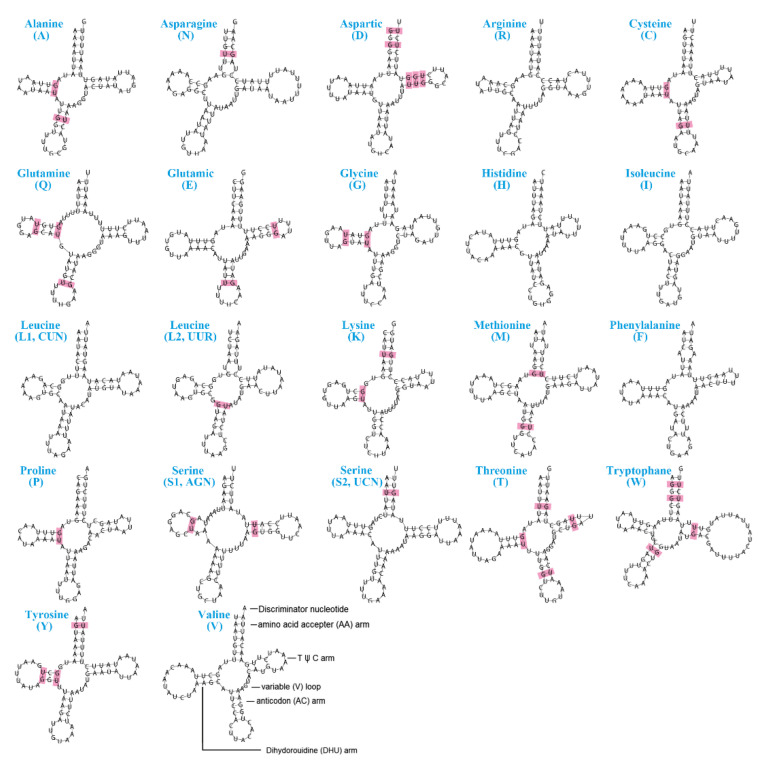
Secondary structures of tRNA genes in the mitogenome of *Haplodiplatys aotouensis*. Mismatched base pairs are indicated by red boxes.

The two *H. aotouensis* mitogenomes both contain the canonical set of 22 tRNA genes and the nucleotide sequences of these genes are pairwise identical (Table 2). These tRNAs range in size from 66 to 79 bp, and the longest tRNA gene is *trnW*. Anticodons of the tRNA genes are identical to other sequenced earwigs. The predicted secondary structures for most of the tRNA genes are typical cloverleaf, whereas the dihydrouridine (DHU) arm of *trnS1* is reduced into a small loop (Figure 4). A total of 37 mismatched base pairs are found in the secondary structure of 16 tRNA genes and all of them are mismatched G-U pairs.

The two rRNA genes, i.e., the large ribosomal RNA (*rrnL*) gene and small ribosomal RNA (*rrnS*) gene are found in the conserved location between *trnL1* and the control region. The *rrnL* gene is 1408 bp in length with an A + T content of 75.4% in both mitogenomes of Fujian’s sample and Jiangxi’s sample. The *rrnS* gene exhibits a slight difference between the two mitogenomes, being 814 bp long in Fujian’s sample and 815 bp long in Jiangxi’s sample. The A + T content of *rrnS* gene is respectively 75.4% and 75.5% in Fujian’s and Jiangxi’s samples.

The two control regions are both shorter than 200 bp. The control region of Fujian’s sample is 139 bp long, with an A + T content of 77%; the control region of Jiangxi’s sample is 197 bp long, with a higher A + T content of 83.8%. Nucleotide sequences of the two control regions are identical on positions 1–93 and 94–139. Three copies of tandem repeats are detected near the 5′ end of both control regions and each copy of repeat comprises eight nucleotides, i.e., TACGCGTA. A subsequent poly-[TA]n stretch is also found near the 3′ end of both control regions but which is 8 bp long (4 TA units) in Fujian’s sample and 66 bp long (33 TA units) in Jiangxi’s sample. The length difference between the two control regions is caused by the different number of TA units.

### 3.5. Phylogenetic Analyses

**Figure 5:**
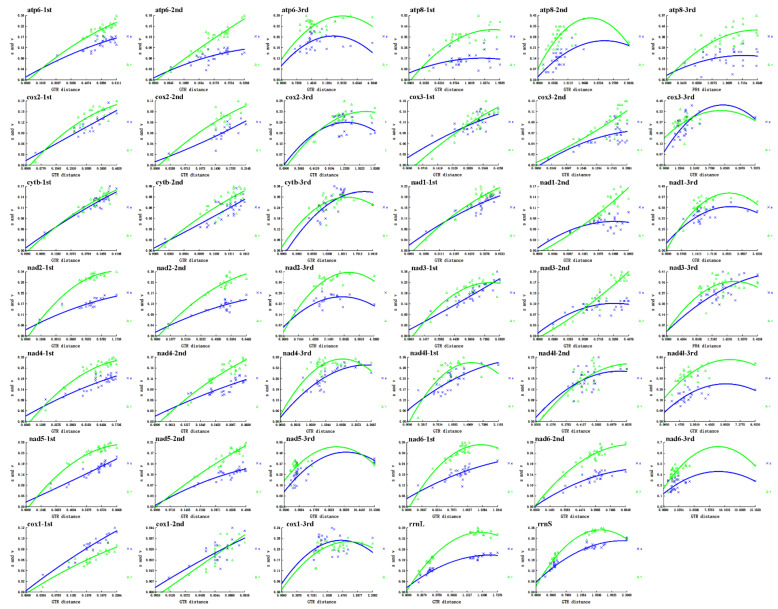
Substitution saturation plots per codon position for PCGs and per gene for rRNA. The plots of *cox1* were calculated excluding the partial *cox1* sequence of *Diplatys flavicollis*.

**Figure 6:**
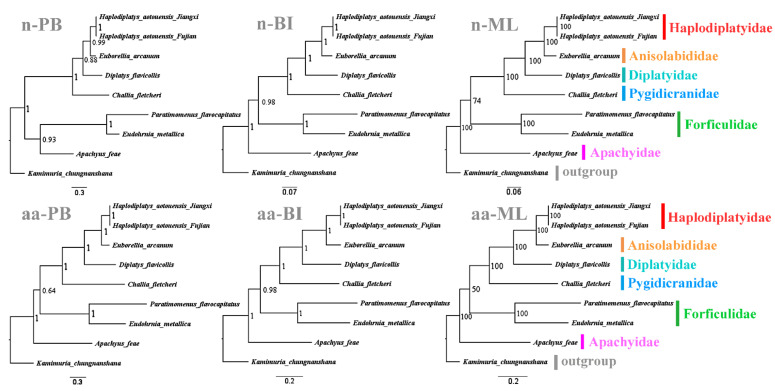
Phylogenetic relationships within Dermaptera inferred from mitogenomic data. Numbers at the nodes are posterior probabilities or bootstrap values. The family names are listed after the species. Abbreviations: n-PB, Phylo–Bayesian tree based on nucleotide dataset; n-BI, Bayesian tree based on nucleotide dataset; n-ML, maximum likelihood tree based on nucleotide dataset; aa-PB, Phylo–Bayesian tree based on amino acid dataset; aa-BI, Bayesian tree based on amino acid dataset; aa-ML, maximum likelihood tree based on amino acid dataset.

To obtain more reliable phylogenetic results, the analysis excluded *cox1*, *cox2*, and *nad2* which are absent from *D. flavicollis* and the saturated genes. According to the saturation plots (Figure 5), *atp8*, *nad4l*, and *nad6*, the third codon positions of all PCGs, and the two rRNA genes are saturated and excluded from the nucleotide dataset. The final nucleotide dataset contains 4542 bases derived from seven PCGs. The amino acid dataset is composed of 2271 amino acids translated from the seven unsaturated PCGs including their third codon positions. Among the six phylogenetic trees (Figure 6), five have identical topological structures. In all trees, the two samples of *H. aotouensis* sequenced in this study are confidently clustered together; Haplodiplatyidae is supported as the sister group of Anisolabididae and their combined clade is grouped with Diplatyidae; Pygidicranidae is basal to the clade comprising Diplatyidae, Anisolabididae, and Haplodiplatyidae. In the PB tree using the nucleotide dataset, Apachyidae is recovered as the sister group of Forficulidae. In the other five trees, Apachyidae is supported as a single basal group of Dermaptera; Forficulidae is the sister group of the clade including Haplodiplatyidae, Anisolabididae, Diplatyidae, and Pygidicranidae.

## 4. Discussion

The two mitogenomes of *H. aotouensis* samples in two geographic areas are almost identical regarding the mitogenome structure but exhibit variations in several PCGs, *rrnS* gene, and noncoding regions. Such intraspecific mitogenomic variation is reasonable for a widespread species but is seldomly studied in earwigs or other related insects. The single variable nucleotide in each of *cox1*, *cox2*, and *nad6* is exclusively restricted to the third codon position and thus did not change the final protein product of these genes. The mutations on the third codon positions are considered neutral because they are usually synonymous with respect to the amino acids [42]. Such frequent mutations of the synonymously variable third codon positions have also been found in other insects [43]. The 1 bp difference in the middle section of the *rrnS* gene might be a random mutation driven by geographical isolation or simply a systematic mistake during the sequencing and assembling processes. The intraspecific difference in the non-coding regions could be attributed to their high rates of nucleotide substitution, insertions or deletions, and the presence of varying copy numbers of tandem repeats [44,45]. As a result, all intraspecific nucleotide differences of *H. aotouensis* did not shift the final protein products.

The mitogenomes of *H. aotouensis* are smaller in size than those of the completely sequenced *Challia fletcheri* Burr, 1904, and *Apachyus feae* de Bormans, 1894 [20,46,47], which is mainly due to the small control region. It is worth noting that control regions of several other earwig mitogenomes failed to be obtained by next-generation sequencing, which might be attributed to the high A and T content and the presence of complicated secondary structures. The standard set of 37 mitochondrial genes with a single control region is recognized in *H. aotouensis* as well as most other sequenced earwigs, whereas the addition or reduction of tRNA genes and control regions have also been reported in other earwigs [20].

The mitochondrial gene arrangement of *H. aotouensis* has apparently diverged from the ancestral pattern. The CREx analysis has been conducted for the earwig mitogenomes to predict the scenarios of rearrangement from the ancestral mitogenome type of *D. yakuba*. The *H. aotouensis* mitogenomes are rearranged from the ancestral mitogenome order by an initial transposition of two tRNA genes, a subsequent reversal of two tRNA genes, and two final TDRL events concerning five tRNA genes. The mitochondrial gene order of *A. feae* changed from *D. yakuba* mainly by four steps of rearrangement events, including the insertion of an additional *trnV*, the transposition of *trnE*, the subsequent reverse transposition of *trnN*, and a final reversal of *trnF* (Figure S1). In *D. flavicollis*, the mitogenome comprises three reversal events and a final TDRL event (Figure S2). In *C. fletcheri*, the mitogenome underwent three transposition events, two reversal events, and a reverse transposition event (Figure S3). The mitogenome of *E. arcanum* includes four reversal events and a final TDRL event (Figure S4). Only in *E. metallica* and *P. flavocapitatus*, the gene order is identical to that of *D. yakuba* [20]. The predicted rearrangement scenarios of earwig mitogenomes, i.e., the number, type, and order of rearrangement events, are different between species that have different gene orders. The scenarios seldomly involve PCGs and cannot change their final arrangement. The multiple rearranged tRNA genes support the prediction of Chen (2022) [20] that extensive mitochondrial gene rearrangement events occur in other unsequenced earwigs and these rearrangements are restricted to tRNA genes.

The RSCU analysis confirms TTA (Leu) as the most frequently used codon, which is similar to most other earwigs [20]. The calculation of Ka/Ks ratios of PCGs reveals a slight difference in *cox1*, *cox2*, and *nad6* between the two geographical samples of *H. aotouensis*, which is due to the presence of a synonymous mutation on the third position of a certain codon in each of the genes. However, the detailed mechanisms underlying these synonymous mutations is unclear. The Ka/Ks values in PCGs also suggests that the fastest evolving PCG is *nd4l* in *H. aotouensis*, instead of *cox1*, *nad2*, or *nad5* as found in other earwigs [20]. Such difference indicates that the evolutionary rates of PCGs are not always consistent between different species. Therefore, an evaluation of evolutionary rates should be conducted before the selection of a single mitochondrial gene for population genetic and phylogenetic research of Dermaptera. The tRNA genes of *H. aotouensis* are the same in the two geographical samples and have identical anticodons with all other sequenced earwigs. However, the tRNA genes are apparently different in nucleotide sequence, size, and location between different species. In the tRNA genes of *H. aotouensis*, the shortened DHU arm of *trnS1* and mismatched G-U base pairs are very common in other earwigs and other metazoans [17,20,48]. The dominance of G-U mismatching might be explained by the bond’s low free energy, which makes it stable and neutral-like [49,50]. The location of the two rRNA genes is identical among all sequenced earwigs but their lengths and nucleotide content are highly variable between species.

In the phylogenetic analyses, the amino acid dataset performed better than the nucleotide dataset by generating identical phylogenetic trees of Dermaptera using different methods. Different types of molecular characters are usually preferred at different taxonomic levels, i.e., the nucleotide dataset is better for phylogenetic reconstruction among closely related taxa, whereas the amino acid dataset performs better to infer deeper phylogenetic relationships [51,52]. The nucleotide dataset can also be used for deeper phylogenetic reconstruction when their saturated third codon positions are excluded [53]. The choice of character type (nucleotide or amino acid) and reconstruction methods (PB, BI, or ML) did not seriously affect the tree topologies of Dermaptera in this study. The addition of the first mitogenomic sequences for Haplodiplatyidae did not change the relationship between the other five families inferred by the most recent mitochondrial phylogenetic study of Chen (2022) [20]. However, the newly sequenced Haplodiplatyidae is herein recovered as the sister group of Anisolabididae instead of Diplatyidae [9]. This result is confusing because Anisolabididae is apparently morphologically diverse from neither Haplodiplatyidae nor Diplatyidae. Although the phylogenetic position of Anisolabididae is also unresolved in previous morphological and molecular studies, it is often grouped with Spongiphoridae or Labiduridae [54]. This confusing clade might be caused by the probable misidentification of *Euborellia arcanum* Matzke & Kočárek, 2015, in Song et al. (2016) [22]. Another possible cause is the inherent deficiency of using mitochondrial DNA without combination with nuclear markers for phylogenetic reconstructions, which has been proved in many other phylogenetic studies [55,56,57]. The basal position of Apachyidae as a sister group to other earwigs was supported in most trees of this study, which is consistent with the phylogenomic study of Wipfler et al. (2020) [54] using nuclear single-copy genes. Apachyidae is also commonly considered a basal lineage in previous studies due to the presence of many primitive characters [4,19,54,58]. Despite the problematic placement of Anisolabididae, the close relationship between Pygidicranidae and Diplatyidae is supported in all trees, which also agrees with the result of Wipfler et al. (2020) [54]. Forficulidae is widely accepted as the most advanced earwig family based on morphology, nuclear genes, and histones [10,19,54,58,59]. However, the mitogenomic data from this study did not support the most advanced position of Forficulidae. The confusing higher-level phylogeny of several clades in this study is mainly attributed to the low suitability of mitochondrial markers alone for deeper phylogenetic reconstruction [57] and the lack of data for many other families of Dermaptera. The investigation of Chen (2022) [20] and this study indeed finds some merit in using mitogenomic data in phylogenetic reconstruction of Dermaptera by recovering partially similar topology with previous studies using both morphological and molecular data. However, the lack of combination with nuclear markers makes mitogenomic data less informative for higher-level phylogeny of Dermaptera, even when the saturated mitochondrial genes and codon positions are excluded and multiple methods are used for the analysis. In addition, many families of Dermaptera have no available mitogenomes, and most families involved in the current mitochondrial phylogenetic analysis have only one sequenced species. The confusing placement of several families in the mitochondrial phylogenetic trees needs reexamination by sequencing more representatives for these families. 

## 5. Conclusions

In conclusion, this study found the intraspecific mitogenomic variation and extensive gene rearrangement of an earwig species from China. However, very few attentions have been paid to improve our understanding of mitogenomic characters of the earwigs. The mitogenomic data is promising in solving the problems in the phylogeny of Dermaptera More comprehensive sampling and sequencing of mitogenomes in combination with nuclear markers in the future are expected to reconstruct a more robust phylogeny of Dermaptera.

## Figures and Tables

**Table 1:** List of species used in this study.

Family	Species	Length (bp)	A + T%	Accession Number	Reference
Haplodiplatyidae	*Haplodiplatys aotouensis* Ma & Chen, 1991 (Fujian)	16,134	71.7	ON186792	this study
	*Haplodiplatys aotouensis* Ma & Chen, 1991 (Jiangxi)	16,222	71.9	ON186793	this study
Apachyidae	*Apachyus feae* de Bormans, 1894	19,029	61.2	MW291948	[20]
Diplatyidae	*Diplatys flavicollis* Shiraki, 1907	12,950	73.5	MW291949	[20]
Pygidicranidae	*Challia fletcheri* Burr, 1904	20,456	72.6	NC_018538	[17]
Anisolabididae	*Euborellia arcanum* Matzke & Kočárek, 2015	16,087	68.3	KX673196	[22]
Forficulidae	*Eudohrnia metallica* (Dohrn, 1865)	16,324	58.7	KX091853	GenBank
	*Paratimomenus flavocapitatus* Shiraki,1906	15,677	67.4	KX091861	GenBank
Perlidae (Plecoptera)	*Kamimuria chungnanshana* Wu, 1938	-	-	NC_028076	[23]

**Table 2:** Mitochondrial genome structure of *Haplodiplatys aotouensis*. Values of Fujian’s (left) and Jiangxi’s (right) samples are separated by semicolons.

Gene	Position (bp)	Size (bp)	Strand	Intergenic Nucleotides	Anticodon	Start/Stop Codons	A + T%
*trnI*	119−188; 119−188	70; 70	J	0; 0	GAT; GAT		72.9; 72.9
*trnM*	200−271; 200−271	72; 72	J	11; 11	CAT; CAT		75.0; 75.0
*nad2*	272−1270; 272−1270	999; 999	J	0; 0		ATT/TAA; ATT/TAA	70.4; 70.4
*trnW*	1310−1388; 1310−1388	79; 79	J	39; 39	TCA; TCA		73.4; 73.4
*cox1*	1446−2945; 1446−2945	1500; 1500	J	57; 57		ATT/TAA; ATT/TAA	65.9; 65.9
*trnL2*	2957−3023; 2957−3023	67; 67	J	11; 11	TAA; TAA		68.7; 68.7
*cox2*	3134−3724; 3134−3724	591; 591	J	110; 110		ATT/TAG; ATT/TAG	67.2; 67.3
*trnK*	3732−3797; 3732−3797	66; 66	J	7; 7	CTT; CTT		63.6; 63.6
*trnD*	3797−3865; 3797−3865	69; 69	J	−1; −1	GTC; GTC		76.8; 76.8
*atp8*	3866−4039; 3866−4039	174; 174	J	0; 0		ATT/TAA; ATT/TAA	72.4; 72.4
*atp6*	4033−4710; 4033−4710	678; 678	J	−7; −7		ATG/TAA; ATG/TAA	70.5; 70.5
*cox3*	4769−5557; 4770−5558	789; 789	J	58; 58		ATG/TAA; ATG/TAA	65.4; 65.4
*trnG*	5598−5665; 5599−5669	68; 68	J	40; 40	TCC; TCC		79.4; 79.4
*nad3*	5666−6019; 5667−6020	354; 354	J	0; 0		ATT/TAA; ATT/TAA	69.8; 69.8
*trnA*	6130−6196; 6139−6205	67; 67	J	110; 118	TGC; TGC		80.6; 80.6
*trnN*	6216−6289; 6225−6298	74; 74	N	19; 19	GTT; GTT		78.4; 78.4
*trnE*	6338−6406; 6349−6417	69; 69	J	48; 50	TTC; TTC		75.4; 75.4
*trnY*	6467−6537; 6478−6548	71; 71	N	60; 60	GTA; GTA		83.1; 83.1
*trnC*	6624−6690; 6635−6701	67; 67	N	86; 86	GCA; GCA		86.6; 86.6
*trnQ*	6700−6768; 6711−6779	69; 69	N	9; 9	TTG; TTG		76.8; 76.8
*trnS1*	6816−6884; 6845−6913	69; 69	J	47; 65	GCT; GCT		72.5; 72.5
*trnR*	6885−6950; 6914−6979	66; 66	J	0; 0	TCG; TCG		74.2; 74.2
*trnF*	6987−7053; 7016−7082	67; 67	J	36; 36	GAA; GAA		83.6; 83.6
*nad5*	7130−8872; 7159−8901	1743; 1743	N	76; 76		ATC/TAA; ATC/TAA	71.1; 71.1
*trnH*	8873−8941; 8902−8970	69; 69	N	0; −3	GTG; GTG		79.7; 79.7
*nad4*	8954−10303; 8983−10332	1350; 1350	N	12; 12		ATG/TAA; ATG/TAA	70.2; 70.2
*nad4l*	10303−10590; 10332−10619	288; 288	N	−1; −1		ATG/TAA; ATG/TAA	71.2; 71.2
*trnT*	10599−10667; 10628−10696	69; 69	J	8; 8	TGT; TGT		76.8; 76.8
*trnP*	10668−10734; 10697−10763	67; 67	N	0; 0	TGG; TGG		76.1; 76.1
*nad6*	10737−11237; 10766−11266	501; 501	J	2; 2		ATT/TAG; ATT/TAG	73.1; 73.3
*c* *ytb*	11270−12409; 11299−12438	1140; 1140	J	32; 32		ATG/TAA; ATG/TAA	69.9; 69.9
*trnS2*	12438−12508; 12467−12537	71; 71	J	28; 28	TGA; TGA		84.5; 84.5
*nad1*	12687−13631; 12716−13660	945; 945	N	178; 178		ATT/TAA; ATT/TAA	66.2; 66.2
*trnL1*	13632−13700; 13661−13729	69; 69	N	0; 0	TAG; TAG		81.2; 81.2
*rrnL*	13701−15108; 13730−15137	1408; 1408	N	0; 0		−	75.4; 75.4
*trnV*	15109−15181; 15138−15210	73; 73	N	0; 0	TAC; TAC		72.6; 72.6
*rrnS*	15182−15995; 15211−16025	814; 815	N	0; 0		−	75.4; 75.5
*CR*	15996−16134; 16026−16222	139; 197	J	0; 0		−	77.0; 83.8

## Data Availability

The data presented in this study are available in NCBI GenBank (Accession number: ON186792 and ON186793).

## Supplementary Materials

The following supporting information can be downloaded at: https://www.mdpi.com/article/10.3390/biology11060807/s1. Figure S1. Reconstruction of mitochondrial gene rearrangement scenarios in the evolution of *Apachyus feae*. Figure S2. Reconstruction of mitochondrial gene rearrangement scenarios in the evolution of *Diplatys flavicollis*. Figure S3. Reconstruction of mitochondrial gene rearrangement scenarios in the evolution of *Challia fletcheri*. Figure S4. Reconstruction of mitochondrial gene rearrangement scenarios in the evolution of *Euborellia arcanum*.
