# Supplementary material for: The First Mitochondrial Genomes of the Family Haplodiplatyidae (Insecta: Dermaptera) Reveal Intraspecific Variation and Extensive Gene Rearrangement
Source: Biology (Basel). 2022 May 25;11(6):807. doi: 10.3390/biology11060807 (PMC9219768; doi:10.3390/biology11060807)
Supplement: Supplementary file 1 [file biology-11-00807-s001.zip › biology-1741041-supplementary.pdf]

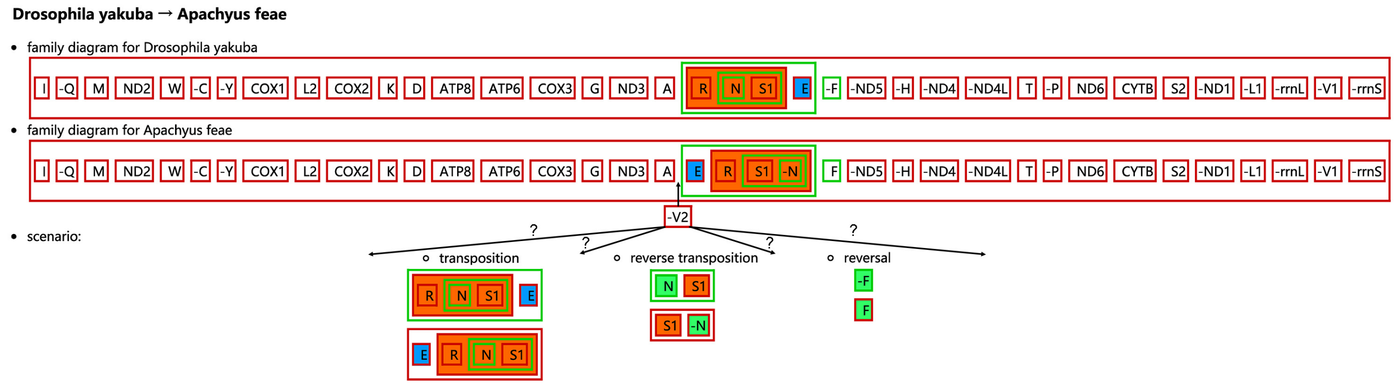

Figure S1. Reconstruction of mitochondrial gene rearrangement scenarios in the evolution of *Apachyus feae*.

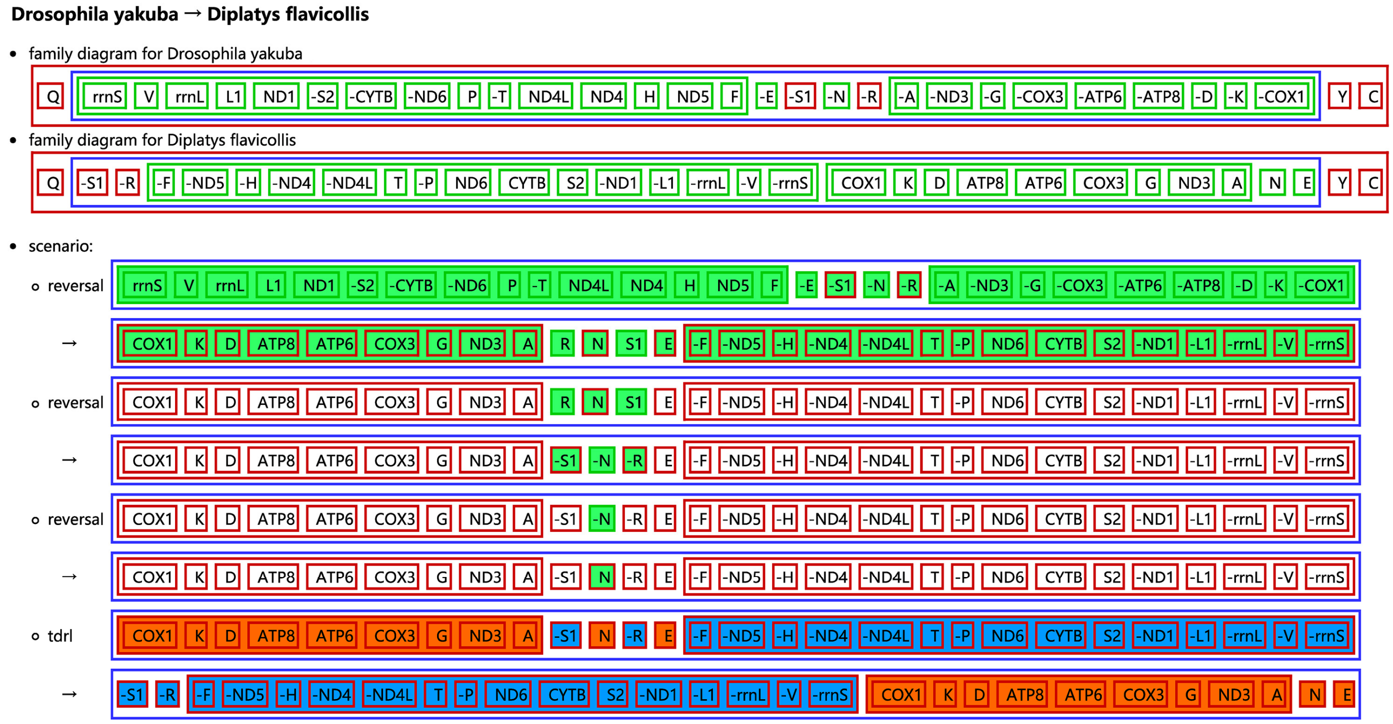

Figure S2. Reconstruction of mitochondrial gene rearrangement scenarios in the evolution of *Diplatys flavicollis*.

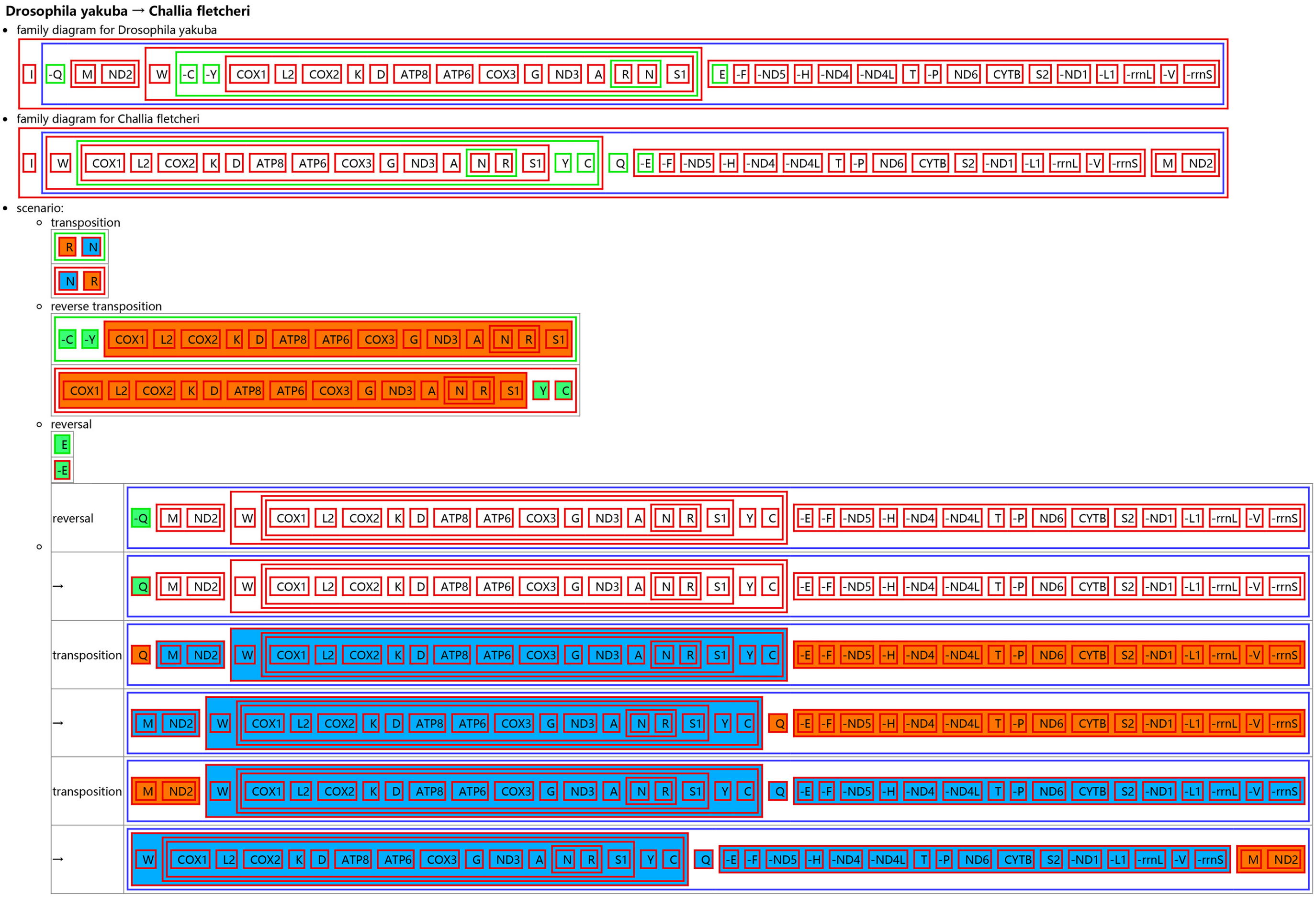

Figure S3. Reconstruction of mitochondrial gene rearrangement scenarios in the evolution of *Challia fletcheri*.

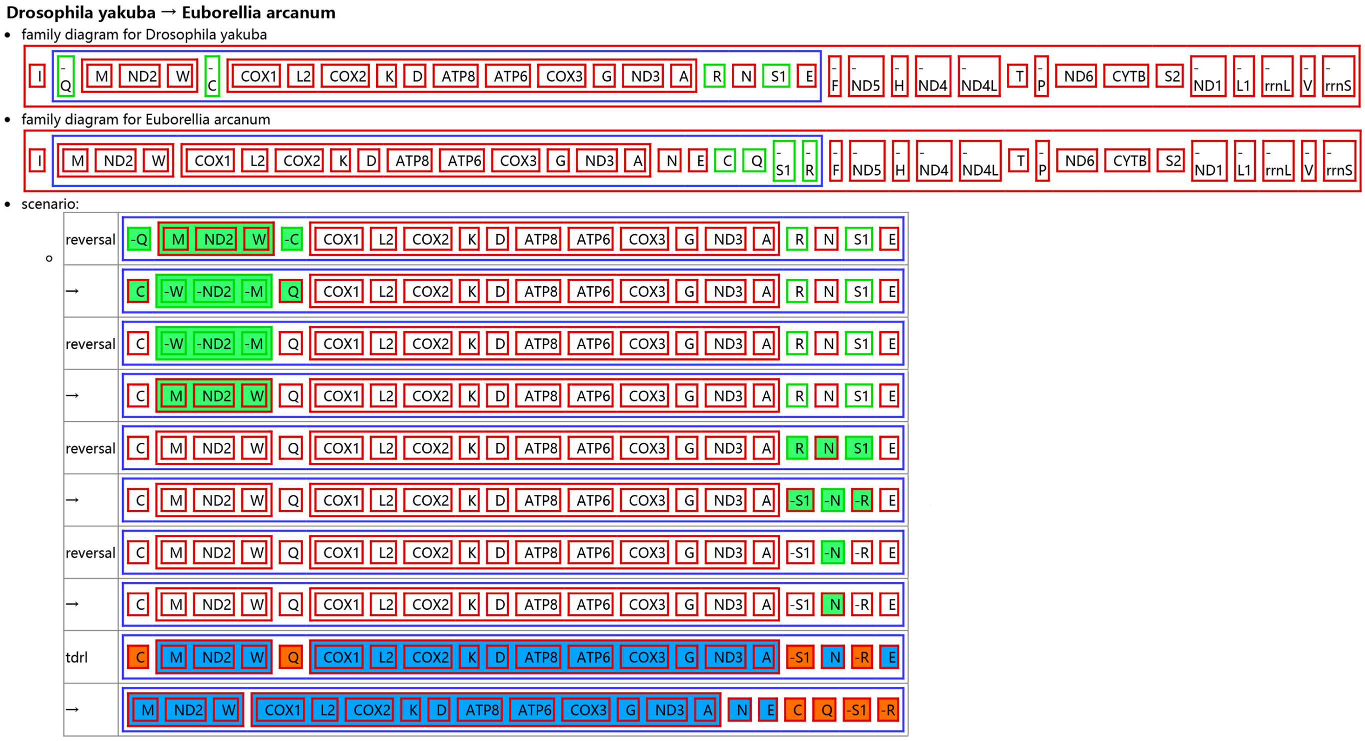

Figure S4. Reconstruction of mitochondrial gene rearrangement scenarios in the evolution of *Euborellia arcanum*.
